# Th17/Treg cell balance in patients with papillary thyroid carcinoma: a new potential biomarker and therapeutic target

**DOI:** 10.3389/fonc.2024.1325575

**Published:** 2024-10-29

**Authors:** Meng-Han Huo, Yilinuer Adeerjiang, Ayiguzhali Abulitipu, Umair Khan, Xin-Xi Li, Lei Zhang, Ye Tian, Sheng Jiang, Can-Can Xu, Xian-Zhen Chao, Ye-Fan Yang, Jin-Xia Zhang, Guo-Li Du

**Affiliations:** ^1^ State Key Laboratory of Pathogenesis, Prevention and Treatment of High Incidence Diseases in Central Asia, Urumqi, China; ^2^ Department of Endocrinology, First Affiliated Hospital of Xinjiang Medical University, Urumqi, China; ^3^ Department of Gastroenterology and Endocrinology, Tianjin Haihe Hospital, Tianjin, China; ^4^ Department of Endocrine Surgery, First Affiliated Hospital of Xinjiang Medical University, Urumqi, Xinjiang, China; ^5^ First Clinical Medical College of Xinjiang Medical University, Urumqi, Xinjiang, China; ^6^ Department of Endocrinology, Bayingolin Mongolian Autonomous Prefecture People's Hospital, Kuerle, China

**Keywords:** Th17, Treg, Th17/Treg homeostasis, papillary thyroid carcinoma (PTC), checkpoint blockade

## Abstract

Papillary thyroid carcinoma (PTC) is the most common subtype of thyroid carcinoma. The most effective treatment for PTC is surgical resection, and patients who undergo surgery have good survival outcomes, but some patients have distant metastasis or even multiorgan metastases at the time of initial diagnosis. Distant metastasis is associated with poorer prognosis and a higher mortality rate. Helper T lymphocyte 17 (Th17) cells and regulatory T lymphocytes (Tregs) play different roles in PTC, and the Th17/Treg balance is closely related to the progression of PTC. Th17 cells play anticancer roles, whereas Tregs play cancer-promoting roles. A Th17/Treg imbalance promotes tumor progression and accelerates invasive behaviors such as tumor metastasis. Th17/Treg homeostasis can be regulated by the TGF‐β/IL‐2 and IL‐6 cytokine axes. Immune checkpoint inhibitors contribute to Treg/Th17 cell homeostasis. For PTC, monoclonal antibodies against CTLA-4, PD-1 and PD-L1 inhibit the activation of Tregs, reversing the Th17/Treg cell imbalance and providing a new option for the prevention and treatment of PTC. This article reviews the role of Tregs and Th17 cells in PTC and their potential targets, aiming to provide better treatment options for PTC.

## Introduction

Thyroid carcinoma is the most common malignant tumor of the head and neck. According to statistics, there were 586,000 patients with thyroid carcinoma worldwide in 2020, and it ranks ninth among malignant tumors worldwide in terms of incidence rate (1). In recent years, the incidence of thyroid carcinoma has increased markedly worldwide (2). In China, the incidence of thyroid carcinoma rapidly increased from 2005 to 2015 (3). Papillary thyroid carcinoma (PTC) is the most common type of thyroid carcinoma, accounting for 90% of new cases (4). Surgical resection is one of the standard treatments for PTC (5). Patients receiving surgical treatment have a better prognosis, and the 10-year survival rate is 93% (6). However, approximately 30%-40% of PTCs metastasize to regional lymph nodes (7, 8), and the presence of cervical lymph node metastasis is related to a poorer prognosis and a lower survival rate (9, 10). The recurrence rate of PTC patients with cervical lymph node metastasis is 3.5 times greater than that of PTC patients without cervical lymph node metastasis (11). However, some patients have distant metastases at the time of initial diagnosis. Toraih EA et al. reported that, of 89,694 PTC patients, 1819 (2%) developed distant metastases at initial diagnosis, 26.3% of whom presented with multiorgan metastases. The most common site of metastasis was the lung (53.4%), followed by the bone (28.1%), liver (8.3%) and brain (4.7%).

The presence of distant metastasis significantly reduces survival in patients with PTC. The 5-year survival rates for patients with bone metastasis and lung metastasis are 25% and 21%, respectively, whereas those for patients with brain and liver metastasis are only 6% and 12%, respectively. Compared with patients with single-organ metastasis, patients with multiorgan metastases have a significantly lower 5-year survival rate (15.3% versus 77.6%) (12). Therefore, there is still a need to discover new molecular markers and therapeutic targets to improve the therapeutic efficacy for PTC patients with distant metastasis and improve patient prognosis and survival. A growing body of research has demonstrated that treatment for thyroid malignancies includes immune checkpoint inhibitors. An increasing number of immune checkpoint inhibitors, including the monoclonal antibodies anti-cytotoxic T lymphocyte antigen 4 (anti-CTLA-4), anti-programmed cell death protein 1 (anti-PD-1), and anti-programmed cell death ligand-1 (anti-PD-L1), have been shown to be effective in the treatment of cancers (13).

## Overview of Th17 cells and tumors

**Figure 1:**
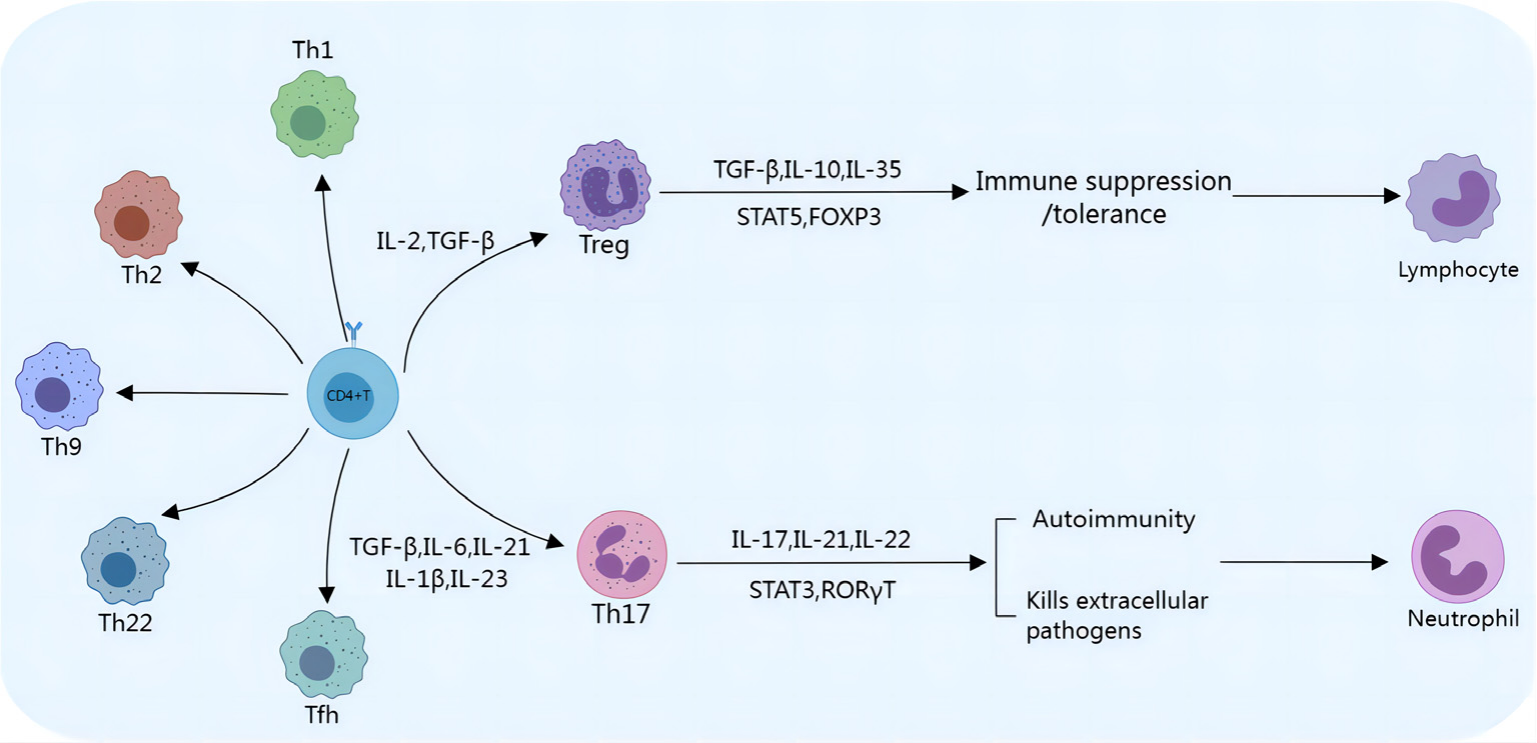
Th17/Treg cell differentiation. Th17/Treg cells differentiate from CD4^+^ T cells. The presence of IL-2 and TGF-β stimulates the initial development of CD4^+^ T cells into Tregs, which express transcription factors such as STAT5 and FoxP3 and secrete cytokines, including TGF-β, IL-10, and IL-35. Tregs play an immunosuppressive/tolerance-promoting role by inhibiting the activation and proliferation of a variety of immune cells, such as NK cells and CD8^+^ T cells. TGF-β, IL-6, and IL-21 promote the development and stabilization of Th17 cells. Th17 cells are most commonly classified by their expression of RORγt and STAT3. Th17 cells can release proinflammatory cytokines to mediate inflammation, inhibit tumor growth, and promote cancer cell apoptosis.

**Figure 2:**
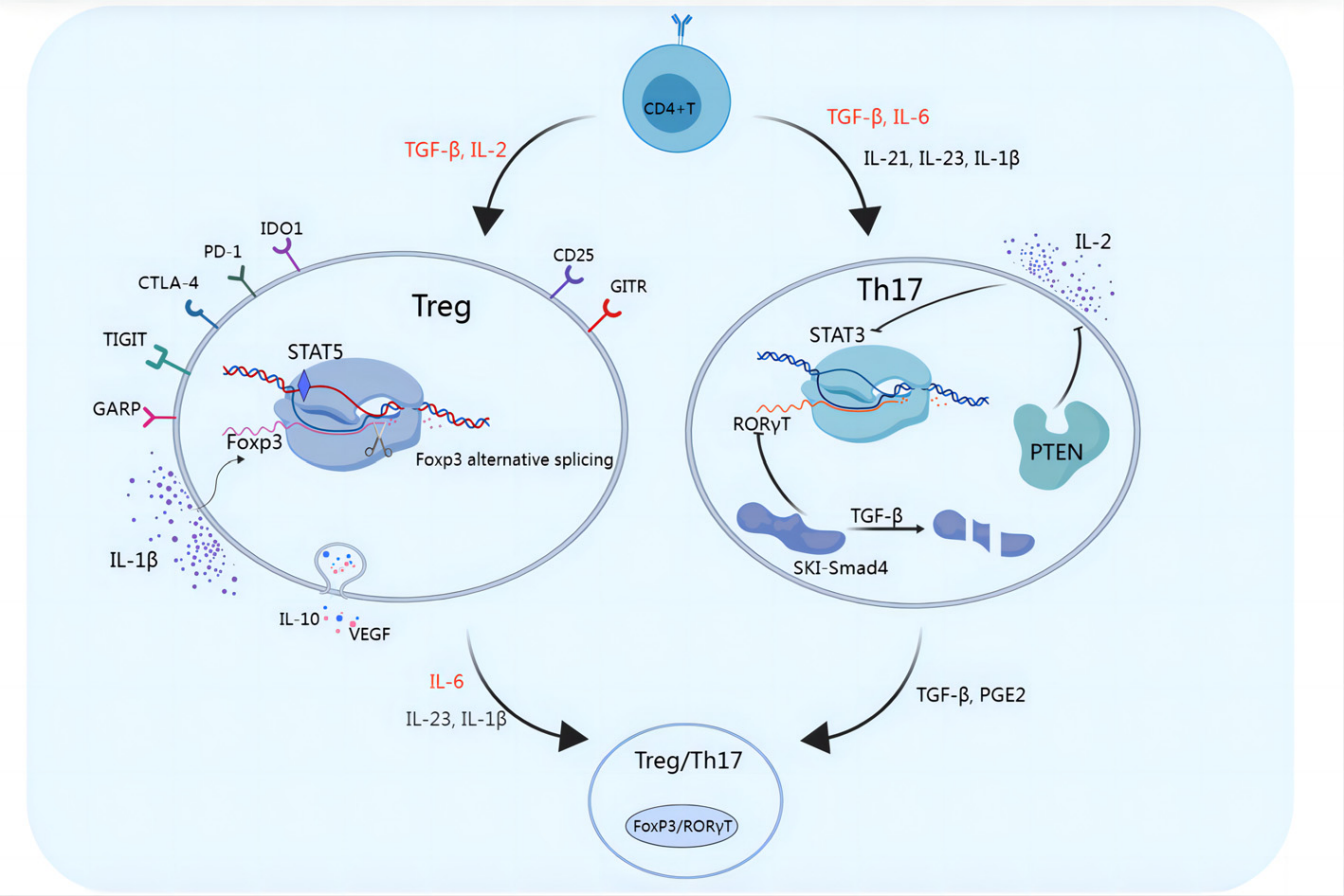
Functional plasticity of Tregs and Th17 cells. Multiple molecules can affect the functional plasticity of Tregs and Th17 cells. TGF-β and IL-2 induce the differentiation of Tregs, which exert immunosuppressive functions and promote immune escape through the secretion of inhibitory cytokines such as IL-10 and VEGF or through the cell-mediated engagement of inhibitory checkpoint molecules such as GITR, PD-1, CTLA-4, TIGIT, CD25, IDO1, and GARP. IL-1β induces alternative splicing of FoxP3, inhibits Treg cell differentiation, and promotes IL-17 production. RORγt is a key transcription factor in Th17 cell development. The SKI-SMAD4 complex inhibits RORγt, in which the SKI protein inhibits acetylation of the Rorc site. TGF-β was shown to modulate the SKI-SMAD4 complex, and in the presence of TGF-β, SKI is degraded, allowing RORγt to be expressed in CD4^+^ T cells and ultimately driving Th17 cell differentiation. IL-2 induces STAT5, reduces STAT3 binding, and inhibits Th17 differentiation. PTEN in Th17 cells inhibits the IL-2 signaling pathway, reduces STAT5 and Treg differentiation, and upregulates STAT3. When FoxP3^+^ Tregs are exposed to IL-6 with or without IL-1β and IL-23, FoxP3 is downregulated, which promotes the expression of Th17 genes, including IL-17, IL-22, IL-23R, and RORγt. TGF-β and PGE2 can also induce Th17-to-Treg cell conversion.

Th17 cells participate in the regulation of the body’s immune system by releasing the proinflammatory cytokine interleukin-17 (IL-17), which promotes the release of inflammatory mediators by epithelial cells, fibroblasts, or macrophages (14, 15). Th17 cells are transformed from CD4^+^ T cells, which depend on interleukin-6 (IL-6), transforming growth factor-β (TGF-β), interleukin-23 (IL-23), interleukin-1β (IL-1β) and interleukin-21 (IL-21), as shown in **Figure 1**. IL-6 and TGF-β are considered key cytokines that induce the transcription factor RAR-related orphan receptor in naive CD4^+^ T cells, which in turn drives Th17 cell differentiation. The SKI-SMAD4 complex inhibits acetylation of the Rorc locus, inhibiting the related orphan receptor γt (RORγt). TGF-β has been shown to degrade the SKI-SMAD4 complex, allowing RORγt to be expressed in CD4^+^ T cells and ultimately driving Th17 cell differentiation (16). A low dose of TGF-β can also inhibit IL-2-mediated signal transduction and activator of transcription 5 (STAT5) activation, inhibiting the differentiation of Tregs and promoting the lineage of Th17 cells (17). Recent studies have also shown that phosphatase and tensin homolog (PTEN) in Th17 cells inhibits the IL-2 signaling pathway and upregulates signal transducer and activator of transcription 3 (STAT3). STAT3 is a transcription factor that supports the Th17 cell pathway and is activated together with TGF-β and IL-6 during T-cell receptor (TCR)/costimulatory signal stimulation, and STAT3 induces the expression of RORγt, which induces Th17 subgroup differentiation (18). TGF-β and IL-6 can also induce IL-23 receptor (IL-23R) expression in Th17 cells (19). IL-23 further maintains the long-term proinflammatory properties of Th17 cells by activating STAT3, RORα, and RORγt in Th17 cells (19, 20). Recent reports have also revealed the role of IL-1β and IL-21 in Th17 cells. IL-1β induces alternative splicing of the transcription factor forkhead box P3 (FoxP3), inhibits Treg differentiation, and promotes IL-17 production (21). IL-21 activates downstream STAT3 and induces Th17 cell differentiation even in the absence of IL-6 (22). Th17 cells produce IL-21, which amplifies the response of Th17 cells and enhances the effect of Th17 cells. IL-21 regulates the function of CD8^+^ T cells through the tumor necrosis factor-α (TNF-α)/IL-17 pathway, mediating tumor regression (**Figure 2**) (23). Ghiringhelli et al. reported that IL-6 and TGF-β induced the polarization of Th17 cells, which express CD39 and CD73, in mice to promote tumor growth. IL-6, IL-1β and IL-23 can induce the expression of CD39 and CD73 in Th17 cells but have no direct effects on tumor growth (24, 25).

Th17 cells, important inflammatory cells, promote the mobilization, recruitment, and activation of neutrophils by regulating the secretion of proinflammatory cytokines, which contributes to the inflammatory response and prevents the immune escape of cancer cells (26, 27). Th17 cells can suppress tumor growth and metastasis and promote cancer cell apoptosis by secreting TNF-α, the soluble dimer cytokine interferon (IFN)-γ, IL-17, IL-21, and IL-22. Th17 cells also recruit macrophages, dendritic cells, granulocytes, natural killer (NK) cells, and CD8^+^ T cells by secreting chemokines and IFN-γ to clear tumors (28). IFN-γ-dependent Th17 cells play important roles in the clearance of tumor cells. In the microenvironment around tumor tissue, Th17-derived cells (ex-Th17 or nonclassical Th1 cells) express more BCL-2, promoting increased IFN-γ expression to facilitate the antitumor immune response (29).

Martin-Orozco reported that Th17 cells promote the activation of tumor-specific CD8^+^ T cells and have an indirect antitumor effect (30). They reported that adoptive T-cell therapy using tumor-specific Th17 cells could generate a strong antitumor immune response, since T cells can recruit dendritic cells into tumor tissues and promote their migration into tumor-draining lymph nodes (31). Research on ovarian cancer patients has shown that tumor-accumulated Th17 cells may play antitumor roles by expressing a variety of cytokines, such as IL-17, inducing helper T lymphocyte 1 (Th1) cell-related chemokines and recruiting T cells to the tumor microenvironment (TME) (32). In breast cancer, IL-22 produced by Th17 cells is associated with reduced tumor formation and a good prognosis (33). In ovarian cancer, the increase in IL-17 produced by Th17 cells improved patient survival (32). Th17-deficient mice are prone to pulmonary melanoma, and adoptive transfer of tumor-specific Th17 cells prevents tumor development (34). Sfanos KS analyzed the correlation between the Th17 cell number in tumor-infiltrating prostate cancer cells and the Gleason grade of prostate cancer and reported that they were negatively correlated (35). Small cell lung cancer (SCLC) is a highly malignant tumor with a tendency to spread. The Th17 cell number in patients with nonmetastatic tumors and primary patients is greater than that in patients with advanced and recurrent disease. The concentration of Th17 cells is also associated with long-term survival in SCLC patients (36). These studies have shown that Th17 cells play antitumor roles. Many research groups have also confirmed that Th17 cell transplantation can lead to tumor regression (34, 37–39).

However, studies have shown that the accumulation of Th17 cells in tumor tissues is associated with a poor prognosis. In lung cancer tissues, Th17 cell accumulation induces epithelial−mesenchymal transition in lung cancer cells, promoting the migration and diffusion of cancer cells through IL-17 (40). By inhibiting tumor cell apoptosis, IL-17 may promote tumor growth and progression and accelerate tumor angiogenesis in some types of cancer (41).

## Overview of Tregs and tumors

Tregs, formerly known as suppressor T cells, are a subset of CD4^+^ T cells that regulate the immune system and are characterized by the expression of FoxP3, which is a specific marker of Treg cells. Tregs play important roles in maintaining self-tolerance and immune system homeostasis, limiting bactericidal immunity and inhibiting antitumor immunity (42). Tregs restrain the activation and proliferation of various immune cells through cytotoxic T lymphocyte antigen 4 (CTLA-4), glucocorticoid-induced tumor necrosis factor receptor (GITR) or the secretion of inhibitory cytokines, such as interleukin-10 (IL-10), TGF-β and interleukin-35 (IL-35), weakening immune function and maintaining the stability of the immune system (43). Other Treg cell surface molecules include programmed cell death protein 1 (PD-1), T-cell immunoreceptor with IG and ITIM domains (TIGIT), indoleamine 2,3-dioxygenase 1 (IDO1), and glycoprotein a repetitions predominant (GARP) but are not unique to Tregs (44). There is an antagonistic relationship between Tregs and Th17 cells, although differentiation is stimulated by similar cytokines (45–47). Tregs and Th17 cells share common precursor cells (naive CD4^+^ T cells), which require a common tumor growth factor (TGF-β) signal to mediate initial differentiation. TGF-β is necessary to induce FoxP3 expression in Tregs and RORγt expression in Th17 cells. Proinflammatory signals regulate the differentiation of these cells. For example, IL-2 and TGF-β induce naive CD4^+^ T cells to differentiate into FoxP3^+^ Tregs, whereas IL-6 and TGF-β induce naive CD4^+^ T cells to differentiate into Th17 cells (45, 46, 48) (**Figure 1**). This feature results in a complex dynamic equilibrium state, coordinating the body’s immune state. As mentioned above, IL-6 and IL-21 induce STAT3 expression, which inhibits the FoxP3 pathway. IL-2 induces STAT5 expression and reduces STAT3 binding, inhibiting Th17 differentiation (19, 49–51). IL-2 supplementation in the treatment of autoimmune diseases may augment Treg cell function and increase self-tolerance of the immune system. As a marker of Tregs with immunosuppressive functions, IL-2Rα (CD25) induces the expression of FoxP3, the master transcriptional regulator that is essential for the development and immune tolerance of Tregs. In mice, CD25 labels a population of CD4^+^ T cells that normalize immune function and prevent lethal autoimmune responses (52, 53). Reconstruction of neonatal thymectomized mice with CD4^+^ CD25^+^ T cells can prevent the occurrence of autoimmune diseases (54). Deficiency of IL-2Rα or IL-2Rβ, or neutralization of IL-2 induces severe autoimmune responses (55–57). As shown in **Figure 2**, Th17/Treg cell homeostasis could be regulated by the TGF-β/IL-2 and IL-6 cytokine axes. Blusten and colleagues reported that the expression of FoxP3 in Tregs was also negatively correlated with the expression of CD127 (IL-7Rα). They reported that the phenotype of CD4^+^CD25^+^CD127^−^ cells typically represents that of human Tregs (58).

In several types of cancers, including ovarian cancer, lung cancer, glioblastoma, non-Hodgkin’s lymphoma, melanoma and other malignancies, Tregs can inhibit the antitumor immune response, promote the development of an immunosuppressive tumor microenvironment, promote immune escape and cancer progression, and decrease the survival rate of patients with cancer (59, 60). The expression of the GARP gene in tumors enhances the activity of TGF-β and induces the differentiation of CD4^+^ T cells into Tregs in the cancer microenvironment, hindering the immune response (61). The presence of Tregs in the tumor microenvironment is associated with advanced stage, invasion, and poor prognosis in patients with malignant tumors (59, 60). The depletion of Treg cells in the tumor microenvironment in mice inhibits the immunosuppression of tumor-infiltrating CD8^+^ T cells, improving the antitumor efficacy of endogenous effector T cells (62).

Tregs are usually enriched in primary tumors, draining lymph nodes and the peripheral blood of cancer patients (63). Recent studies have shown that increased numbers of Tregs are associated with tumor progression and poor prognosis (64). The accumulation of FoxP3^+^ Tregs, especially a relatively high ratio of Tregs: T effector cells (Teffs), is often associated with a poor prognosis in many tumors, including ovarian cancer (65, 66), lung cancer (67), glioblastoma (68), melanoma and other malignancies (69, 70). The role of Tregs in immune escape has been demonstrated in many clinical studies and *in vitro* studies. For example, in patients with advanced melanoma, transient Treg cell depletion leads to the regression of metastatic lesions (71). In breast cancer patients, pretreatment with Treg cells is associated with an antitumor immune response and improved clinical symptoms (72). In patients with metastatic breast cancer, Treg depletion followed by cancer antigen inoculation produces effective antitumor CD4^+^ T and CD8^+^ T cells (73).

## Tregs and PTC

Inflammation is thought to be associated with thyroid carcinoma, indicating that immune cells play important roles in the pathogenesis of PTC. There are many immune cells and their mediators in the microenvironment of thyroid carcinoma. The infiltration of immune cells in the PTC microenvironment is closely related to tumor progression. M2 macrophages, dendritic cells, mast cells, neutrophils, and myeloid-derived suppressor cells play tumor-promoting roles in PTC, whereas CD8^+^ T cells, NK cells and helper T lymphocyte 9 (Th9) cells play antitumor roles in PTC (13).

**Figure 3:**
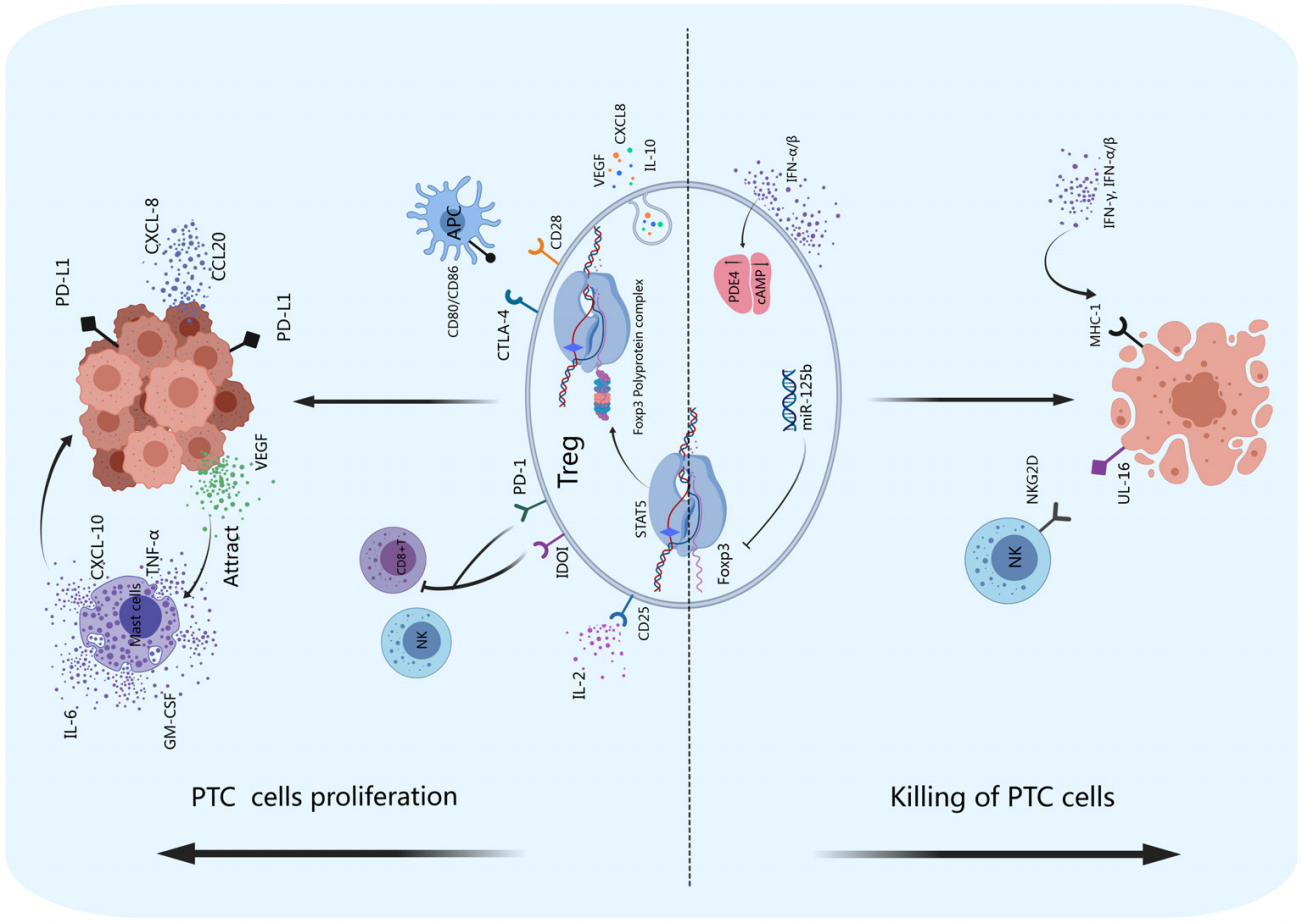
Tregs and PTC. Tregs exert immunosuppressive functions by producing inhibitory cytokines such as IL-10, CXCL8, and VEGF and promote tumor angiogenesis. The inhibitory receptors IDOI and PD-1 on the surface of Tregs can inhibit the activity of NK cells and CD8^+^ T cells, promoting immune escape. PD-I can also bind to PD-L1 on the surface of PTCs to promote the proliferation of tumor cells. CD28 and CTLA-4 can competitively bind to CD80/CD86 on the surface of APCs, thereby enhancing the inhibitory function of Tregs. FoxP3 can maintain the inhibitory effect of Tregs on the immune system by forming a 400–800 kDa multiprotein complex with its transcription partners. Thyroid cancer cells can produce VEGF, recruit mast cells to infiltrate thyroid cancer tissue, and stimulate mast cells to produce IL-6, TNF-α, GM-CSF and CXCL-10, accelerating tumor growth. Thyroid cancer cells can also release CCL20 and CXCL8, which promote PTC invasion and metastasis *in vitro*. The *miR-125b* gene negatively regulates the expression of FoxP3 and promotes autophagy in thyroid cancer. IFN-α/β can increase the expression of PDE4 in Tregs, suppress the production of cAMP, and promote the apoptosis of thyroid tumor cells. NKG2D on the surface of NK cells can bind to UL-16 on the surface of thyroid cancer cells and cause the apoptosis of tumor cells.

As shown in **Figure 3**, Tregs play roles in maintaining the tolerance of thyroid carcinoma. FoxP3 is a key regulatory gene for the development of Tregs that inhibits the antitumor immune response by producing immunosuppressive molecules, such as IL-10, CTLA-4 and PD-1, and releasing vascular endothelial growth factor (VEGF) to stimulate tumor angiogenesis (74, 75). Tregs can also promote antitumor inhibitory activity by inhibiting CD8^+^ T cells (76). A lower intratumoral CD8^+^/FoxP3^+^ ratio was found in PTC patients with the BRAFV600E mutation, and it was associated with high expression levels of the immunosuppressive molecules arginase-1, IDO1 and programmed cell death ligand-1 (PD-L1) (77). In addition, an increase in the number of Tregs in the metastatic lymph nodes of thyroid carcinoma patients is associated with increased incidence of invasive thyroid carcinoma (78). FoxP3^+^ Tregs were found in PTC tumor tissues, and their infiltration was associated with disease stage and lymph node metastasis (79). Compared with that in simple nodular goiter tissue, the FoxP3^+^ Treg cell number in PTC tumor tissue is greater, and it is positively correlated with disease stage (80). Yu et al. reported that, compared with that in patients with nontoxic multinodular goiter (MNG), the number of FoxP3^+^ Tregs in thyroid tissue and peripheral blood was significantly greater in PTC patients. Szyberg et al. reported that the FoxP3^+^ Treg cell number was greater in PTC patients than in MNG patients (81, 82). Ugolini et al. (83) reported that Treg cells can be detected in PTC patients (up to 43%). In addition, increased infiltration of FoxP3^+^ Treg cells was associated with lymph node metastasis, extrathyroidal infiltration, and multifocality in papillary thyroid microcarcinoma (PTMC) (84). French et al. reported that the frequency of FoxP3^+^ Treg cells was associated with lymph node metastasis in 110 PTC patients (79). An increase in the number of FoxP3+ Tregs was associated with late TNM stage and PTC lymph node metastasis (79, 80). Zeng et al. (85) reported that high expression of FoxP3 in the tumor microenvironment of PTC patients was significantly greater than that in patients with lower expression. These findings suggest that high expression of FoxP3 in PTC patients may promote lymph node metastasis. Cunha et al. (86) reported that nuclear FoxP3 staining in differentiated thyroid cancer cells was stronger in young patients and those with lymph node metastasis than in the corresponding control groups, indicating that FoxP3 expression might be associated with thyroid cancer aggressiveness.

Studies have also shown that the expression of FoxP3 in thyroid carcinoma cells is significantly related to the drug resistance phenotype of PTC to radioactive iodine therapy (83). Zhongqin Gong reported that inhibiting the expression of FoxP3 induced the apoptosis of thyroid carcinoma cells and suppressed their proliferation and migration (87). The expression of FoxP3 in tumor cells likely contributes to carcinogenesis. Some molecules, including nuclear factor of activated T cells (NFAT) (88) and runt-related transcription factor 1 (RUNX1) (89), can bind to the promoter region of the FOXP3 gene and activate the differentiation of Tregs. FOXP3 overexpression in Treg cells can promote tumor cell growth in non-small cell lung cancer (NSCLC). FOXP3 was found to regulate CD4^+^/CD25^+^ or CD4^+^/CD25^−^ Treg cell development and function (90–92). CD4^+^/CD25^+^/FOXP3^+^ Treg cells suppress the immune system and promote tumor progression by reducing the anticancer immunity of CD4^+^ T or CD8^+^ effector T cells. The activation of Tregs reduces antipathogenic or anticancer immunity, leading to cancer progression and infection (93). In addition, the miR-125b gene negatively regulates the expression of FoxP3, promotes autophagy in thyroid carcinoma, and enhances the therapeutic effect of cisplatin (94). Therefore, FoxP3 may be an important intervention target for thyroid carcinoma treatment.

How do Tregs play roles in the development of PTC? Studies have confirmed that to sustain the inhibitory effect of Tregs in the immune system, FoxP3 cooperates with its transcription partners. Rudra D reported that FoxP3 forms a 400–800 kDa multiprotein complex and identified 361 related proteins. FoxP3 regulates approximately 30% of the proteins in this complex at the transcriptional level (95). FoxP3 can interact with most of the cofactors of the complex and promote each other. A number of key molecules have been identified through continuous research, such as NFAT (88), runt-related transcription factor (RUNX) (96), GATA transcription factor 3 (GATA3) (97) and the transcription factor forkhead box P1 (FoxP1) (98).

## Th17 cells and PTC

Th17 cells exert antitumor effects through the secretion of IL-17 (99, 100). Xie et al. reported that IL-17 can exert antitumor effects by inhibiting tumor angiogenesis (101). In a tumor model in IL-17-deficient mice, tumor growth and distant metastasis were accelerated, suggesting that IL-17 has an antitumor effect (31). Compared with that in healthy controls, the concentration of Th17 cells in the peripheral blood and tissue of PTC patients is greater (99). The Th17 cell concentration in peripheral blood is positively correlated with the level of serum IL-17, but negatively correlated with tumor size (99). In PTC, the infiltration of Th17 cells and the expression of the transcription factor RORγt are negatively correlated with lymph node metastasis (102).

**Figure 4:**
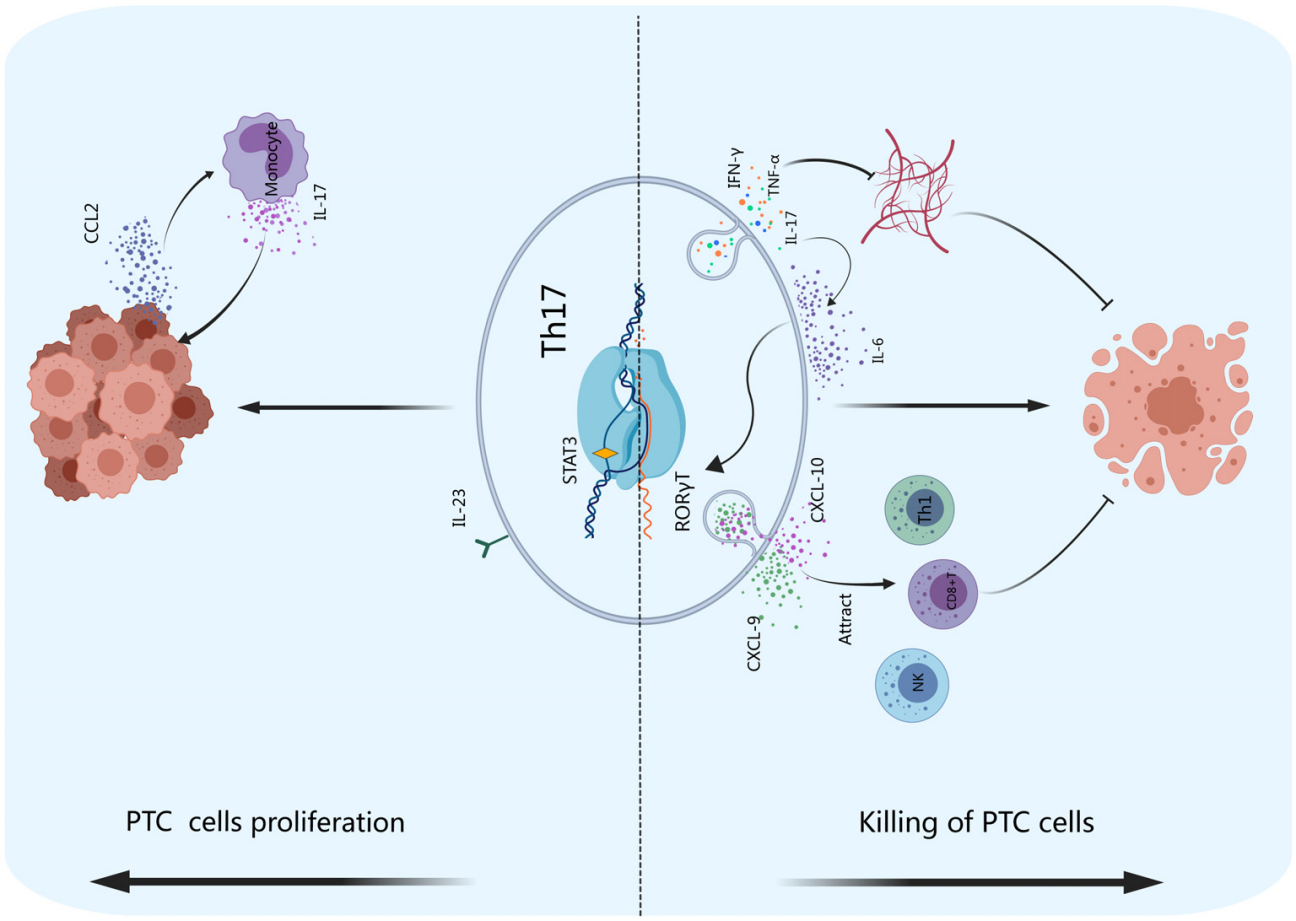
Th17 cells and PTC. Th17 cells exert indirect antitumor effects by secreting chemokines such as CXCL-9 and CXCL-10 to attract effector cells such as Th1 and CD8^+^ T cells and NK cells to accumulate and kill tumor cells in tumor tissues. Th17 cells can also inhibit the formation of tumor blood vessels and promote PTC cell apoptosis by producing IFN-γ, TNF-α and IL-17. IL-17 can enhance the effect of IL-6, transforming CD4^+^ T cells into Th17 cells. PTC cells can produce CCL2, which stimulates monocytes to produce IL-17, promoting tumor cell proliferation.

The antitumor effect of Th17 cells occurs through the recruitment of immune cells and the secretion of specific cytokines rather than through killing tumor cells directly (32). Th17 cells lack the expression of cytotoxicity-related molecules, such as granzymes and perforin. As shown in **Figure 4**, Th17 cells attract Th1 cells, CD8^+^ T cells, NK cells, and other effector cells to accumulate in tumor tissues and kill tumor cells through the secretion of chemokines such as CXCL-9 and CXCL-10 (103). In the tumor microenvironment, the infiltration of Th17 cells has been observed to be positively correlated with that of immune effector cells (including CD8^+^ T cells and NK cells), and the infiltration of these cells promotes cytotoxic cell-mediated antitumor responses (32, 104). In the thyroid carcinoma microenvironment (105), NK cells kill thyroid carcinoma cells by expressing the activation receptor NKG2D, which binds to its ligand UL16 expressed on thyroid carcinoma cells (106). On the other hand, Th17 cells inhibit tumor angiogenesis by secreting TNF-α, IFN-γ, IL-17F, IL-21 and IL-22, which inhibit tumor growth and promote the apoptosis of cancer cells (28). IL-17 produced by Th17 cells can also induce IL-6 secretion, thereby activating the STAT3 signaling pathway (107). Th17 cells also strongly induce the expression of CCL20 in tumor tissue, stimulating the mobilization of dendritic cells and other leukocytes to the tumor site, where they activate CD8^+^ T cells to suppress tumor growth (107). The Th17 profile, which includes immune cells, cytokines, chemokines, and their receptors, has protective effects, serving as a trigger for the clearance of thyroid tumors (108).

The lymphocyte infiltration rate of PTC patients was greater than that of patients with benign thyroid lesions. However, PTC combined with thyroiditis is associated with a better prognosis (109, 110). Poorly differentiated thyroid carcinoma and anaplastic thyroid carcinoma have poor prognoses. Compared with that in PTC, the infiltration of lymphocytes in these two thyroid carcinomas is significantly reduced, indicating that these lymphocytes may exhibit protective functions in thyroid carcinoma (111). However, Galano Carvalho reported that the protein expression of IL-17 in benign lesions (follicular thyroid adenoma and goiter) was lower than that in malignant lesions (differentiated thyroid carcinoma and medullary thyroid carcinoma) (112). Therefore, Th17 cells may play roles in promoting tumorigenesis and development. IL-17 induces the expression of vascular endothelial growth factor, which in turn induces TGF-β, promoting tumor growth and metastasis (113–115). Tumor cells can stimulate IL-17 secretion by monocytes through the expression of C-C motif ligand 2 (CCL2), and further promote inflammation and tumor growth (116, 117).

## The plasticity and balance of Th17 cells and Tregs

In the past 20 years, it has been believed that the precursor cells derived from the differentiation of naive CD4^+^ T cells are irreversible. However, during the cell polarization process, although different T-cell subsets express different genes and have different regulatory characteristics, each subset retains significant developmental plasticity. Th17 cells are distributed mainly in the intestine, lung, skin, and other barrier sites and are highly plastic. The plasticity relationship between Th17 cells and Tregs has also been gradually recognized (118–120). Increasing evidence has shown that Th17 cells and Tregs have greater developmental plasticity than other cell subsets do. As FoxP3+ Tregs are exposed to IL-6 (with or without IL-1β and IL-23), FoxP3 expression is downregulated in favor of the expression of Th17 cell genes, including IL-17, IL-22, IL-23R, and RORγt (121). Th17 cells can also be converted into Tregs under certain conditions (122). This conversion may be affected by TGF-β and prostaglandin E2 (PGE2) (122) (**Figure 2**). Phillips reported that Tregs expressed ROR-γt and IL-17 in the peripheral blood of patients with NSCLC (123). Voo also reported that IL-17-producing cells can simultaneously express FoxP3 and ROR-γt (124). Although they transform into Th17 cells, Tregs can continuously express ROR-γt and secrete IL-17 *in vitro*. Memory Tregs that secrete IL-17 have the same phenotypic and functional characteristics as traditional Th17 cells do (125). Th17 cells that accumulate in melanoma, breast cancer, ovarian cancer, and colon cancer can express FoxP3 (126). Th17 cells cloned from T lymphocytes in human tumor tissues can differentiate into FoxP3^+^ Tregs and repeatedly stimulate T-cell receptors *in vitro* (127). These studies provide evidence that Th17 cells that accumulate in tumors can differentiate into Tregs.

In recent years, the role of the balance of Th17/Treg cells in autoimmune diseases and tumors has attracted much attention. A large amount of evidence shows that the Th17/Treg cell balance is the basis of the pathogenesis of autoimmune diseases (128, 129). Correction of this imbalance has been used as a strategy for the treatment of several diseases, including rheumatoid arthritis (RA), psoriasis, psoriatic arthritis, ankylosing spondylitis, systemic lupus erythematosus (SLE), multiple sclerosis (MS) and inflammatory bowel disease (IBD) (128, 129). Monoclonal antibodies have been shown to be effective at neutralizing Th17-related cytokines, including IL-6, TNF-α, IL-17, and IL-23 (128, 129). Monoclonal antibodies against human IL-6R, tocilizumab and sarilumab increase the concentration of Treg cells, reducing the number of Th17 cells in RA patients and affecting the proportion of Th17/Treg cells (130–132).

The balance of Th17/Treg cells affects the development and metastasis of cancer. An imbalance of Th17/Treg cells contributes to the immune escape of tumor cells and is closely related to the stage of the tumor and the poor prognosis of patients (28, 36, 44, 133, 134). Under normal physiological conditions, Th17 cells and Tregs inhibit the differentiation and function of each other and maintain a dynamic balance, maintaining immune self-stability. The Th17/Treg cell ratio in the peripheral blood of patients with NSCLC, renal cell carcinoma (RCC), endometrial carcinoma (EC), and cervical cancer is significantly greater than that in the peripheral blood of healthy controls (135–140). In other cancers, such as epithelial ovarian cancer (EOC), pancreatic cancer (PC), tongue squamous cell carcinoma (TSCC), hepatocellular carcinoma (HCC), and oral squamous cell carcinoma (OSCC), the ratio of Th17/Treg cell is low (134, 141–144). Huo et al. reported that as the number of Treg cells in the spinal cord of a bone cancer pain (BCP) mouse model increased, the Th17 cell number increased, resulting in Th17/Treg cell imbalance, which promoted the activation of microglia and the development of BCP (145). Wu et al. reported that the number of Th17 cells in the peripheral blood of a lung cancer mouse model was significantly reduced, whereas the number of Treg cells was increased, resulting in tumor-associated immunosuppression (146). Lin et al. reported that a disrupted Th17/Treg cell balance was attributed to the progression of cervical cancer (UCC) and that this imbalance was reversed after treatment (138, 139). These studies suggest that the Th17/Treg cell balance plays very important roles in the development of cancer. Reversing the Th17/Treg cell imbalance could be an effective therapeutic strategy for treating tumors.

## Checkpoint blockade

**Figure 5:**
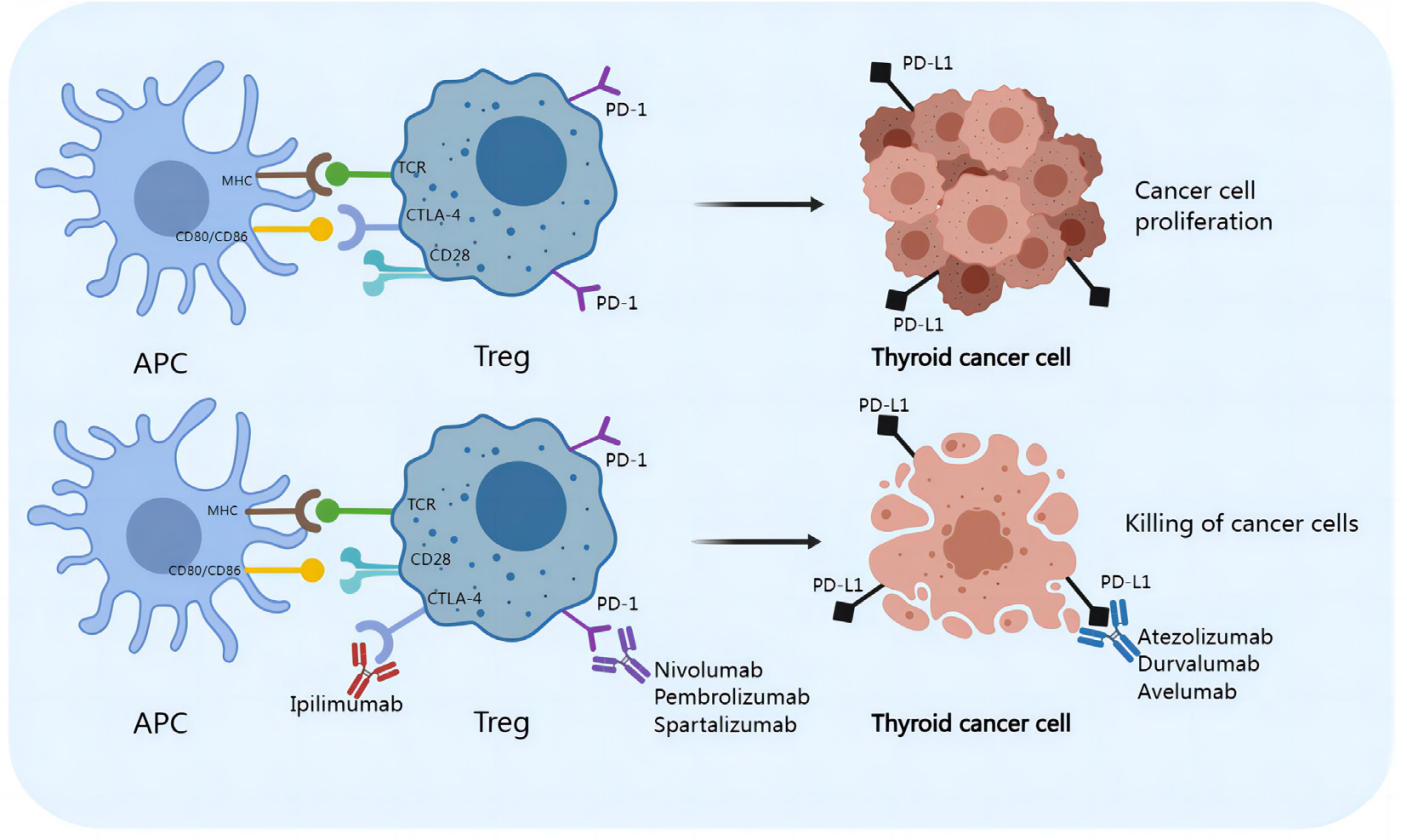
Checkpoint blockades reverse the Th17/Treg imbalance and improve the treatment of PTC.

Malignancy is accompanied by a high mortality rate. Surgical resection is one of the standard treatments for PTC. Patients who receive surgical treatment have a better prognosis than those who do not. However, metastasis, such as cervical lymph node metastasis or site-specific metastasis, including lung, bone, liver and brain metastasis, indicates a poor prognosis and low survival rates in PTC patients (12). Therefore, traditional surgical excision treatment has limited feasibility. Treatments for thyroid malignances include immune checkpoint inhibitors (shown in **Figure 5**) in addition to surgical resection. Inhibition of inhibitory receptors (checkpoints) on Tregs with monoclonal antibodies can inhibit melanoma, bladder cancer, breast cancer, renal cell carcinoma, ovarian cancer, lung cancer, and colorectal cancer (147). An increasing number of immune checkpoint inhibitors, including monoclonal anti-CTLA-4, anti-PD-1, and anti-PD-L1 antibodies, have been shown to be effective in the treatment of cancers. During the progression of PTC, neoantigens can be captured by APCs and MHC-II molecules on the surface of APCs, which can bind to TCRs on Tregs. The activation of Tregs requires a costimulatory signal transmitted by CD28, which is activated by binding to CD80 and/or CD86 on the APC. Tumor cells upregulate CTLA-4 on Tregs, and CTLA-4 competes with CD28 for binding to CD80/CD86 on APCs. The interaction of CTLA-4 with CD80/CD86 leads to inhibitory signal transduction in Tregs, which favors the proliferation of thyroid carcinoma cells (148). The immunosuppressive activity of CTLA-4 is mediated by downregulating Th17 cells and enhancing Tregs, which can disrupt Treg/Th17 homeostasis. In addition, tumor cells express high levels of PD-L1 and/or PD-L2, which bind to PD-1 on Tregs, resulting in inhibitory signals (149). Monoclonal antibodies targeting CTLA-4 (e.g., ipilimumab), PD-1 (including atezolizumab, pembrolizumab, and spartalizumab) and PD-L1 (e.g., atezolizumab, durvalumab, and avelumab) inhibit the interaction of CTLA-4/CD80/86 and PD-1/PD-L1, respectively, and inhibit the activation of Tregs, reversing the Th17/Treg cell imbalance. However, the roles of Th17 cells or other IL-17-producing immune cells in the mechanism of checkpoint blockade therapy have not been adequately described (44). The combination of surgical resection with immune checkpoint suppression therapy is expected to be widely used in future clinical practice, especially for aggressive PTCs, providing a new option for the prevention and treatment of PTC.

The extent of surgical lymph node dissection and the use of adjuvant radioiodine therapy are still highly debated in the management of patients with PTC. The identification of novel prognostic markers would be helpful in predicting the risk of disease recurrence and aggressiveness (78). Th1 polarization, characterized by the production of interferon (IFN)-γ and the activation of cytotoxic CD8^+^ T cells, is known to promote tumor elimination (150). A clinical trial assessing the efficacy of the PD-1-blocking antibody MDX-1106 in cancer therapy showed promising results with minimal adverse effects (151). In recent years, different genetic alterations in molecular pathways that determine the development and progression of PTC have been identified (BRAF mutations, TERT promoter mutations, RAS mutations, RET/PTC gene rearrangements and RET mutations). As small organic compounds, tyrosine kinase inhibitors (TKIs) inhibit tyrosine kinase autophosphorylation and activation. Most of them are multikinase inhibitors that act on the above reported molecular pathways. Clarification of the factors involved in PTC progression, including tumor growth, angiogenesis, and local and distant metastasis, have provided new therapeutic directions for aggressive PTC (152, 153). The development of new drugs is promising because of the absence of specific and effective drugs for the treatment of aggressive PTC.

## Summary

In conclusion, Th17 cells and Tregs play different roles in the development of PTC, and their balance is closely related to the progression and invasive characteristics of PTC. Th17 cells play anticancer roles, whereas Tregs play cancer-promoting roles. This imbalance affects tumor progression and invasive behaviors such as tumor metastasis, and the TGF-β/IL-2 and IL-6 cytokine axes greatly contribute to Th17/Treg homeostasis. At present, molecular targeted therapies for Th17 cells and Tregs have been approved for the treatment of inflammatory diseases, such as RA, psoriasis, psoriatic arthritis, ankylosing spondylitis, systemic lupus erythematosus, MS, and IBD. Th17/Treg cells are involved in the pathogenesis of PTC. Immunotherapy against Th17/Treg cells has good potential in PTC and is worthy of further study.

## Glossary

PTC: papillary thyroid carcinoma
Th17: helper T lymphocyte 17
Treg: regulatory T lymphocyte
IL-2: interleukin-2
TGF-β: transforming growth factor-β
STAT5: signal transducer and activator of transcription 5
FoxP3: factor forkhead box P3
IL-10: interleukin-10
IL-35: interleukin-35
NK: natural killer
IL-6: interleukin-6
IL-21: interleukin-21
RORγt: related orphan receptor γt
STAT3: signal transducer and activator of transcription 3
TCR: T cell receptor
VEGF: vascular endothelial growth factor
GITR: glucocorticoid-induced tumor necrosis factor receptor
PD-1: programmed cell death protein1
CTLA-4: cytotoxic T lymphocyte antigen 4
TIGIT: T Cell Immunoreceptor with Ig and ITIM Domains
IDO1: indoleamine 2,3-dioxygenase 1
GARP: glycoprotein a repetitions predominant
IL-1β: interleukin-1β
IL-17: interleukin-17
IL-23: interleukin-23
IL-22: interleukin-22
PGE2: prostaglandin Eion 5
PTEN: phosphatase 2
CXCL: C-X-C motif ligand
PD-L1: programmath ligand-1
APC: antigen-presenting ed cell decell
Teff cell: T effector cell
TNF-α: tumor necrosis factor-α
GM-CSF: granulocyte-macrophage colony-stimulating factor
IFN: interferon
Th1: helper T lymphocyte 1
TME: tumor microenvironment
ECM: extracellular matrix
Th9: helper T lymphocyte 9
PDE4: phosphodiesterase 4
MNG: multinodular goiter
PDC: plasmacytoid dendritic cells
PTMC: papillary thyroid microcarcinoma
MHC-I: major histocompatibility complex-1
VEGFR: vascular endothelial growth factor receptor
Camp: cyclic AMP
CCL2: C-C motif ligand 2
NSCLC: non-small cell lung cancer
SLE: systemic lupus erythematosus
MS: multiple sclerosis
IBD: inflammatory bowel disease
RCC: renal cell carcinoma
EC: endometrial carcinoma
EOC: epithelial ovarian cancer
PC: pancreatic cancer
TSCC: tongue squamous cell carcinoma
HCC: hepatocellular carcinoma
OSCC: oral squamous cell carcinoma
BCP: bone cancer pain
MTAbs: monoclonal antibodies
anti-CLA-4: anti-cytotoxic T lymphocyte antigen 4
anti-PD-1: anti-programmed cell death protein-1
anti-PD-L1: anti-programmed cell death ligand-1

## References

[1] Sung H Ferlay J Siegel RL Laversanne M Soerjomataram I Jemal A. Global cancer statistics 2020: GLOBOCAN estimates of incidence and mortality worldwide for 36 cancers in 185 countries. CA Cancer J Clin. (2021) 71:209–49. doi: 10.3322/caac.21660 33538338

[2] Chen AY Jemal A Ward EM. Increasing incidence of differentiated thyroid cancer in the United States, 1988-2005. Cancer. (2009) 115:3801–7. doi: 10.1002/cncr.v115:16 19598221

[3] Wang J Yu F Shang Y Ping Z Liu L. Thyroid cancer: incidence and mortality trends in China, 2005-2015. Endocrine. (2020) 68:163–73. doi: 10.1007/s12020-020-02207-6 32002755

[4] Cabanillas ME McFadden DG Durante C. Thyroid cancer. Lancet. (2016) 388:2783–95. doi: 10.1016/S0140-6736(16)30172-6 27240885

[5] Haugen BR Alexander EK Bible KC Doherty GM Mandel SJ Nikiforov YE. American thyroid association management guidelines for adult patients with thyroid nodules and differentiated thyroid cancer: the american thyroid association guidelines task force on thyroid nodules and differentiated thyroid cancer. Thyroid. (2015) 2016:26(1). doi: 10.1089/thy.2015.0020 PMC4739132 26462967

[6] Hundahl SA Fleming ID Fremgen AM Menck HR. A National Cancer Data Base report on 53,856 cases of thyroid carcinoma treated in the U.S., 1985-1995 [see commetns. Cancer. (1998) 83:2638–48. doi: 10.1002/(SICI)1097-0142(19981215)83:12<2638::AID-CNCR31>3.0.CO;2-1 9874472

[7] Boone RT Fan C-Y Hanna EY. Well-differentiated carcinoma of the thyroid. Otolaryngol Clin North Am. (2003) 36(1):73–90. doi: 10.1016/S0030-6665(02)00127-5 12803010

[8] Baek S-K Jung K-Y Kang S-M Kwon S-Y Woo J-S Cho S-H. Clinical risk factors associated with cervical lymph node recurrence in papillary thyroid carcinoma. Thyroid. (2010) 20:147–52. doi: 10.1089/thy.2008.0243 19785522

[9] Adam MA Pura J Goffredo P Dinan MA Reed SD Scheri RP. Presence and number of lymph node metastases are associated with compromised survival for patients younger than age 45 years with papillary thyroid cancer. J Clin Oncol. (2015) 33:2370–5. doi: 10.1200/JCO.2014.59.8391 26077238

[10] Zaydfudim V Feurer ID Griffin MR Phay JE. The impact of lymph node involvement on survival in patients with papillary and follicular thyroid carcinoma. Surgery. (2008) 144(6):1070–7. doi: 10.1016/j.surg.2008.08.034 19041020

[11] Dong W Horiuchi K Tokumitsu H Sakamoto A Noguchi E Ueda Y. Time-varying pattern of mortality and recurrence from papillary thyroid cancer: lessons from a long-term follow-up. Thyroid. (2019) 29:802–8. doi: 10.1089/thy.2018.0128 30931815

[12] Toraih EA Hussein MH Zerfaoui M Attia AS Marzouk Ellythy A Mostafa A. Site-specific metastasis and survival in papillary thyroid cancer: the importance of brain and multi-organ disease. Cancers (Basel). (2021) 13(7):1625. doi: 10.3390/cancers13071625 33915699 PMC8037301

[13] Varricchi G Loffredo S Marone G Modestino L Fallahi P Ferrari SM. The immune landscape of thyroid cancer in the context of immune checkpoint inhibition. Int J Mol Sci. (2019) 20(16):3934. doi: 10.3390/ijms20163934 31412566 PMC6720642

[14] Shao S He F Yang Y Yuan G Zhang M Yu X. Th17 cells in type 1 diabetes. Cell Immunol. (2012) 280:16–21. doi: 10.1016/j.cellimm.2012.11.001 23246831

[15] Aggarwal S Gurney AL. IL-17: prototype member of an emerging cytokine family. J Leukoc Biol. (2002) 71:1–8. doi: 10.1189/jlb.71.1.1 11781375

[16] Zhang S Takaku M Zou L Gu A-D Chou W-C Zhang G. Reversing SKI-SMAD4-mediated suppression is essential for TH17 cell differentiation. Nature. (2017) 551:105–9. doi: 10.1038/nature24283 PMC5743442 29072299

[17] Qin H Wang L Feng T Elson CO Niyongere SA Lee SJ. TGF-beta promotes Th17 cell development through inhibition of SOCS3. J Immunol. (2009) 43(3):97–105. doi: 10.1016/j.cyto.2008.07.161 PMC2851540 19535626

[18] Kim HS Jang SW Lee W Kim K Sohn H Hwang SS. PTEN drives Th17 cell differentiation by preventing IL-2 production. J Exp Med. (2017) 214:3381–98. doi: 10.1084/jem.20170523 PMC5679178 29018045

[19] Yang XO Panopoulos AD Nurieva R Chang SH Wang D Watowich SS. STAT3 regulates cytokine-mediated generation of inflammatory helper T cells. J Biol Chem. (2007) 282:9358–63. doi: 10.1074/jbc.C600321200 17277312

[20] O'Shea JJ Paul WE. Mechanisms underlying lineage commitment and plasticity of helper CD4+ T cells. Science. (2010) 327(5969):1098–102. doi: 10.1126/science.1178334 PMC2997673 20185720

[21] Mailer RKW Joly A-L Liu S Elias S Tegner J Andersson J. IL-1β promotes Th17 differentiation by inducing alternative splicing of FOXP3. Sci Rep. (2015) 5:14674. doi: 10.1038/srep14674 26441347 PMC4593960

[22] Korn T Bettelli E Gao W Awasthi A Jäger A Strom TB. IL-21 initiates an alternative pathway to induce proinflammatory T(H)17 cells. Nature. (2007) 448:484–7. doi: 10.1038/nature05970 PMC3805028 17581588

[23] Phan-Lai V Dang Y Gad E Childs J Disis ML. The antitumor efficacy of IL2/IL21-cultured polyfunctional neu-specific T cells is TNFα/IL17 dependent. Clin Cancer Res. (2016) 22:2207–16. doi: 10.1158/1078-0432.CCR-15-2273 PMC4854769 26660518

[24] Ghiringhelli F Bruchard M Chalmin F Rébé C. Production of adenosine by ectonucleotidases: a key factor in tumor immunoescape. J BioMed Biotechnol. (2012) 2012:473712. doi: 10.1155/2012/473712 23133312 PMC3481458

[25] Chalmin F Mignot G Bruchard M Chevriaux A Végran F Hichami A. Stat3 and Gfi-1 transcription factors control Th17 cell immunosuppressive activity via the regulation of ectonucleotidase expression. Immunity. (2012) 36:362–73. doi: 10.1016/j.immuni.2011.12.019 22406269

[26] Tesmer LA Lundy SK Sarkar S Fox DA. Th17 cells in human disease. Immunol Rev. (2008) 223:87–113. doi: 10.1111/j.1600-065X.2008.00628.x 18613831 PMC3299089

[27] Wang Y Xing F Ye S Xiao J Di J Zeng S. Jagged-1 signaling suppresses the IL-6 and TGF-β treatment-induced Th17 cell differentiation via the reduction of RORγt/IL-17A/IL-17F/IL-23a/IL-12rb1. Sci Rep. (2015) 5:8234. doi: 10.1038/srep08234 25648768 PMC4316398

[28] Fu L-Q Yang X Cai M-H Yao J-Y Jin W-W Mou Y-P. Role of treg/th17 imbalance, microbiota and miRNAs in pancreatic cancer: therapeutic options. Crit Rev Immunol. (2020) 40:75–92. doi: 10.1615/CritRevImmunol.2020033631 32421980

[29] Basdeo SA Cluxton D Sulaimani J Moran B Canavan M Orr C. Ex-th17 (Nonclassical th1) cells are functionally distinct from classical th1 and th17 cells and are not constrained by regulatory T cells. J Immunol. (2017) 198:2249–59. doi: 10.4049/jimmunol.1600737 28167631

[30] Martin-Orozco N Muranski P Chung Y Yang XO Yamazaki T Lu S. T helper 17 cells promote cytotoxic T cell activation in tumor immunity. Immunity. (2009) 31:787–98. doi: 10.1016/j.immuni.2009.09.014 PMC2787786 19879162

[31] Kryczek I Wei S Szeliga W Vatan L Zou W. Endogenous IL-17 contributes to reduced tumor growth and metastasis. Blood. (2009) 114:357–9. doi: 10.1182/blood-2008-09-177360 PMC2714210 19289853

[32] Kryczek I Banerjee M Cheng P Vatan L Szeliga W Wei S. Phenotype, distribution, generation, and functional and clinical relevance of Th17 cells in the human tumor environments. Blood. (2009) 114:1141–9. doi: 10.1182/blood-2009-03-208249 PMC2723011 19470694

[33] Horlock C Stott B Dyson PJ Morishita M Coombes RC Savage P. The effects of trastuzumab on the CD4+CD25+FoxP3+ and CD4+IL17A+ T-cell axis in patients with breast cancer. Br J Cancer. (2009) 100:1061–7. doi: 10.1038/sj.bjc.6604963 PMC2670001 19277040

[34] Muranski P Boni A Antony PA Cassard L Irvine KR Kaiser A. Tumor-specific Th17-polarized cells eradicate large established melanoma. Blood. (2008) 112:362–73. doi: 10.1182/blood-2007-11-120998 PMC2442746 18354038

[35] Sfanos KS Bruno TC Maris CH Xu L Thoburn CJ DeMarzo AM. Phenotypic analysis of prostate-infiltrating lymphocytes reveals TH17 and Treg skewing. Clin Cancer Res. (2008) 14:3254–61. doi: 10.1158/1078-0432.CCR-07-5164 PMC3082357 18519750

[36] Koyama K Kagamu H Miura S Hiura T Miyabayashi T Itoh R. Reciprocal CD4+ T-cell balance of effector CD62Llow CD4+ and CD62LhighCD25+ CD4+ regulatory T cells in small cell lung cancer reflects disease stage. Clin Cancer Res. (2008) 14:6770–9. doi: 10.1158/1078-0432.CCR-08-1156 18980970

[37] Muranski P Borman ZA Kerkar SP Klebanoff CA Ji Y Sanchez-Perez L. Th17 cells are long lived and retain a stem cell-like molecular signature. Immunity. (2011) 35:972–85. doi: 10.1016/j.immuni.2011.09.019 PMC3246082 22177921

[38] Paulos CM Carpenito C Plesa G Suhoski MM Varela-Rohena A Golovina TN. The inducible costimulator (ICOS) is critical for the development of human T(H)17 cells. Sci Transl Med. (2010) 2:55ra78. doi: 10.1126/scitranslmed.3000448 PMC6282816 20980695

[39] Kryczek I Zhao E Liu Y Wang Y Vatan L Szeliga W. Human TH17 cells are long-lived effector memory cells. Sci Transl Med. (2011) 3:104ra0. doi: 10.1126/scitranslmed.3002949 PMC3345568 21998407

[40] Salazar Y Zheng X Brunn D Raifer H Picard F Zhang Y. Microenvironmental Th9 and Th17 lymphocytes induce metastatic spreading in lung cancer. J Clin Invest. (2020) 130:3560–75. doi: 10.1172/JCI124037 PMC7324180 32229721

[41] Sharp SP Avram D Stain SC Lee EC. Local and systemic Th17 immune response associated with advanced stage colon cancer. J Surg Res. (2017) 208:180–6. doi: 10.1016/j.jss.2016.09.038 PMC6086724 27993206

[42] Li C Yuan J Zhu Y-F Yang X-J Wang Q Xu J. Imbalance of th17/treg in different subtypes of autoimmune thyroid diseases. Cell Physiol Biochem. (2016) 40:245–52. doi: 10.1159/000452541 27855396

[43] Ballke C Gran E Baekkevold ES Jahnsen FL. Characterization of regulatory T-cell markers in CD4+ T cells of the upper airway mucosa. PloS One. (2016) 11:e0148826. doi: 10.1371/journal.pone.0148826 26866695 PMC4751285

[44] Knochelmann HM Dwyer CJ Bailey SR Amaya SM Elston DM Mazza-McCrann JM. When worlds collide: Th17 and Treg cells in cancer and autoimmunity. Cell Mol Immunol. (2018) 15:458–69. doi: 10.1038/s41423-018-0004-4 PMC6068176 29563615

[45] Bettelli E Carrier Y Gao W Korn T Strom TB Oukka M. Reciprocal developmental pathways for the generation of pathogenic effector TH17 and regulatory T cells. Nature. (2006) 441:235–8. doi: 10.1038/nature04753 16648838

[46] Veldhoen M Hocking RJ Atkins CJ Locksley RM Stockinger B. TGFbeta in the context of an inflammatory cytokine milieu supports *de novo* differentiation of IL-17-producing T cells. Immunity. (2006) 24:179–89. doi: 10.1016/j.immuni.2006.01.001 16473830

[47] Tato CM O'Shea JJ. Immunology: what does it mean to be just 17? Nature. (2006) 441:166–8. doi: 10.1038/441166a 16688162

[48] Zhou L Ivanov II Spolski R Min R Shenderov K Egawa T. IL-6 programs T(H)-17 cell differentiation by promoting sequential engagement of the IL-21 and IL-23 pathways. Nat Immunol. (2007) 8:967–74. doi: 10.1038/ni1488 17581537

[49] Laurence A Tato CM Davidson TS Kanno Y Chen Z Yao Z. Interleukin-2 signaling via STAT5 constrains T helper 17 cell generation. Immunity. (2007) 26:371–81. doi: 10.1016/j.immuni.2007.02.009 17363300

[50] Ichiyama K Yoshida H Wakabayashi Y Chinen T Saeki K Nakaya M. Foxp3 inhibits RORgammat-mediated IL-17A mRNA transcription through direct interaction with RORgammat. J Biol Chem. (2008) 283:17003–8. doi: 10.1074/jbc.M801286200 18434325

[51] Yang X-P Ghoreschi K Steward-Tharp SM Rodriguez-Canales J Zhu J Grainger JR. Opposing regulation of the locus encoding IL-17 through direct, reciprocal actions of STAT3 and STAT5. Nat Immunol. (2011) 12:247–54. doi: 10.1038/ni.1995 PMC3182404 21278738

[52] Takahashi T Kuniyasu Y Toda M Sakaguchi N Itoh M Iwata M. Immunologic self-tolerance maintained by CD25+CD4+ naturally anergic and suppressive T cells: induction of autoimmune disease by breaking their anergic/suppressive state. Int Immunol. (1998) 10:1969–80. doi: 10.1093/intimm/10.12.1969 9885918

[53] Sakaguchi S Sakaguchi N Asano M Itoh M Toda M. Immunologic self-tolerance maintained by activated T cells expressing IL-2 receptor alpha-chains (CD25). Breakdown of a single mechanism of self-tolerance causes various autoimmune diseases. J Immunol. (1995) 155:1151–64. doi: 10.4049/jimmunol.155.3.1151 7636184

[54] Asano M Toda M Sakaguchi N Sakaguchi S. Autoimmune disease as a consequence of developmental abnormality of a T cell subpopulation. J Exp Med. (1996) 184:387–96. doi: 10.1084/jem.184.2.387 PMC2192701 8760792

[55] Malek TR Yu A Vincek V Scibelli P Kong L. CD4 regulatory T cells prevent lethal autoimmunity in IL-2Rbeta-deficient mice. Implications for the nonredundant function of IL-2. Immunity. (2002) 17:167–78. doi: 10.1016/S1074-7613(02)00367-9 12196288

[56] Willerford DM Chen J Ferry JA Davidson L Ma A Alt FW. Interleukin-2 receptor alpha chain regulates the size and content of the peripheral lymphoid compartment. Immunity. (1995) 3:521–30. doi: 10.1016/1074-7613(95)90180-9 7584142

[57] Setoguchi R Hori S Takahashi T Sakaguchi S. Homeostatic maintenance of natural Foxp3(+) CD25(+) CD4(+) regulatory T cells by interleukin (IL)-2 and induction of autoimmune disease by IL-2 neutralization. J Exp Med. (2005) 201:723–35. doi: 10.1084/jem.20041982 PMC2212841 15753206

[58] Liu W Putnam AL Xu-Yu Z Szot GL Lee MR Zhu S. CD127 expression inversely correlates with FoxP3 and suppressive function of human CD4+ T reg cells. J Exp Med. (2006) 203:1701–11. doi: 10.1084/jem.20060772 PMC2118339 16818678

[59] Tang Y Xu X Guo S Zhang C Tang Y Tian Y. An increased abundance of tumor-infiltrating regulatory T cells is correlated with the progression and prognosis of pancreatic ductal adenocarcinoma. PloS One. (2014) 9:e91551. doi: 10.1371/journal.pone.0091551 24637664 PMC3956642

[60] Shou J Zhang Z Lai Y Chen Z Huang J. Worse outcome in breast cancer with higher tumor-infiltrating FOXP3+ Tregs: a systematic review and meta-analysis. BMC Cancer. (2016) 16:687. doi: 10.1186/s12885-016-2732-0 27566250 PMC5002190

[61] Metelli A Wu BX Fugle CW Rachidi S Sun S Zhang Y. Surface expression of TGFβ Docking receptor GARP promotes oncogenesis and immune tolerance in breast cancer. Cancer Res. (2016) 76:7106–17. doi: 10.1158/0008-5472.CAN-16-1456 PMC5504525 27913437

[62] Budhu S Schaer DA Li Y Toledo-Crow R Panageas K Yang X. Blockade of surface-bound TGF-β on regulatory T cells abrogates suppression of effector T cell function in the tumor microenvironment. Sci Signal. (2017) 10(494):eaak9702. doi: 10.1126/scisignal.aak9702 28851824 PMC5851440

[63] Núñez NG Tosello Boari J Ramos RN Richer W Cagnard N Anderfuhren CD. Tumor invasion in draining lymph nodes is associated with Treg accumulation in breast cancer patients. Nat Commun. (2020) 11:3272. doi: 10.1038/s41467-020-17046-2 32601304 PMC7324591

[64] Whiteside TL Schuler P Schilling B. Induced and natural regulatory T cells in human cancer. Expert Opin Biol Ther. (2012) 12:1383–97. doi: 10.1517/14712598.2012.707184 PMC3730844 22849383

[65] Curiel TJ Coukos G Zou L Alvarez X Cheng P Mottram P. Specific recruitment of regulatory T cells in ovarian carcinoma fosters immune privilege and predicts reduced survival. Nat Med. (2004) 10:942–9. doi: 10.1038/nm1093 15322536

[66] Leffers N Gooden MJM de Jong RA Hoogeboom B-N ten Hoor KA Hollema H. Prognostic significance of tumor-infiltrating T-lymphocytes in primary and metastatic lesions of advanced stage ovarian cancer. Cancer Immunol Immunother. (2009) 58:449–59. doi: 10.1007/s00262-008-0583-5 PMC11030692 18791714

[67] Tao H Mimura Y Aoe K Kobayashi S Yamamoto H Matsuda E. Prognostic potential of FOXP3 expression in non-small cell lung cancer cells combined with tumor-infiltrating regulatory T cells. Lung Cancer. (2012) 75(1):95–101. doi: 10.1016/j.lungcan.2011.06.002 21719142

[68] Sayour EJ McLendon P McLendon R De Leon G Reynolds R Kresak J. Increased proportion of FoxP3+ regulatory T cells in tumor infiltrating lymphocytes is associated with tumor recurrence and reduced survival in patients with glioblastoma. Cancer Immunol Immunother. (2015) 64:419–27. doi: 10.1007/s00262-014-1651-7 PMC4774199 25555571

[69] deLeeuw RJ Kost SE Kakal JA Nelson BH. The prognostic value of FoxP3+ tumor-infiltrating lymphocytes in cancer: a critical review of the literature. Clin Cancer Res. (2012) 18:3022–9. doi: 10.1158/1078-0432.CCR-11-3216 22510350

[70] Shang B Liu Y Jiang S-j Liu Y. Prognostic value of tumor-infiltrating FoxP3+ regulatory T cells in cancers: a systematic review and meta-analysis. Sci Rep. (2015) 5:15179. doi: 10.1038/srep15179 26462617 PMC4604472

[71] Rasku MA Clem AL Telang S Taft B Gettings K Gragg H. Transient T cell depletion causes regression of melanoma metastases. J Transl Med. (2008) 6:12. doi: 10.1186/1479-5876-6-12 18334033 PMC2330026

[72] Ladoire S Arnould L Apetoh L Coudert B Martin F Chauffert B. Pathologic complete response to neoadjuvant chemotherapy of breast carcinoma is associated with the disappearance of tumor-infiltrating foxp3+ regulatory T cells. Clin Cancer Res. (2008) 14:2413–20. doi: 10.1158/1078-0432.CCR-07-4491 18413832

[73] Rech AJ Mick R Martin S Recio A Aqui NA Powell DJ. CD25 blockade depletes and selectively reprograms regulatory T cells in concert with immunotherapy in cancer patients. Sci Transl Med. (2012) 4:134ra62. doi: 10.1126/scitranslmed.3003330 PMC4425934 22593175

[74] Wolf D Sopper S Pircher A Gastl G Wolf AM. Treg(s) in cancer: friends or foe? J Cell Physiol. (2015) 230:2598–605. doi: 10.1002/jcp.25016 25913194

[75] Facciabene A Peng X Hagemann IS Balint K Barchetti A Wang L-P. Tumour hypoxia promotes tolerance and angiogenesis via CCL28 and T(reg) cells. Nature. (2011) 475:226–30. doi: 10.1038/nature10169 21753853

[76] Barbi J Pardoll D Pan F. Treg functional stability and its responsiveness to the microenvironment. Immunol Rev. (2014) 259:115–39. doi: 10.1111/imr.2014.259.issue-1 PMC3996455 24712463

[77] Angell TE Lechner MG Jang JK LoPresti JS Epstein AL. MHC class I loss is a frequent mechanism of immune escape in papillary thyroid cancer that is reversed by interferon and selumetinib treatment in vitro. Clin Cancer Res. (2014) 20:6034–44. doi: 10.1158/1078-0432.CCR-14-0879 PMC4252612 25294906

[78] French JD Kotnis GR Said S Raeburn CD McIntyre RC Klopper JP. Programmed death-1+ T cells and regulatory T cells are enriched in tumor-involved lymph nodes and associated with aggressive features in papillary thyroid cancer. J Clin Endocrinol Metab. (2012) 97:E934–E43. doi: 10.1210/jc.2011-3428 PMC3387418 22466343

[79] French JD Weber ZJ Fretwell DL Said S Klopper JP Haugen BR. Tumor-associated lymphocytes and increased FoxP3+ regulatory T cell frequency correlate with more aggressive papillary thyroid cancer. J Clin Endocrinol Metab. (2010) 95:2325–33. doi: 10.1210/jc.2009-2564 PMC2869546 20207826

[80] Gogali F Paterakis G Rassidakis GZ Kaltsas G Liakou CI Gousis P. Phenotypical analysis of lymphocytes with suppressive and regulatory properties (Tregs) and NK cells in the papillary carcinoma of thyroid. J Clin Endocrinol Metab. (2012) 97:1474–82. doi: 10.1210/jc.2011-1838 22399513

[81] Yu H Huang X Liu X Jin H Zhang G Zhang Q. Regulatory T cells and plasmacytoid dendritic cells contribute to the immune escape of papillary thyroid cancer coexisting with multinodular non-toxic goiter. Endocrine. (2013) 44:172–81. doi: 10.1007/s12020-012-9853-2 23264145

[82] Szylberg Ł Bodnar M Harasymczuk J Marszalek A. Expression of FoxP3 protein plays a key role in thyroid tumors in children. Fetal Pediatr Pathol. (2014) 33:84–91. doi: 10.3109/15513815.2013.864347 24328999

[83] Ugolini C Elisei R Proietti A Pelliccioni S Lupi C Borrelli N. FoxP3 expression in papillary thyroid carcinoma: a possible resistance biomarker to iodine 131 treatment. Thyroid. (2014) 24:339–46. doi: 10.1089/thy.2012.0589 23915122

[84] Ryu HS Park YS Park HJ Chung YR Yom CK Ahn S-H. Expression of indoleamine 2,3-dioxygenase and infiltration of FOXP3+ regulatory T cells are associated with aggressive features of papillary thyroid microcarcinoma. Thyroid. (2014) 24:1232–40. doi: 10.1089/thy.2013.0423 24742251

[85] Zeng R Lyu Y Niu H Yang K Yan X. FoxP3 promotes lymph node metastasis in patients with papillary thyroid carcinoma complicated with Hashimoto's thyroiditis. Transl Cancer Res. (2020) 9:1337–50. doi: 10.21037/tcr.2020.01.12 PMC8797984 35117482

[86] Cunha LL Morari EC Nonogaki S Soares FA Vassallo J Ward LS. Foxp3 expression is associated with aggressiveness in differentiated thyroid carcinomas. Clinics (Sao Paulo). (2012) 67:483–8. doi: 10.6061/clinics/2012(05)13 PMC3351250 22666793

[87] Chu R Liu SYW Vlantis AC van Hasselt CA Ng EKW Fan MD. Inhibition of Foxp3 in cancer cells induces apoptosis of thyroid cancer cells. Mol Cell Endocrinol. (2015) 399:228–34. doi: 10.1016/j.mce.2014.10.006 25312920

[88] Wu Y Borde M Heissmeyer V Feuerer M Lapan AD Stroud JC. FOXP3 controls regulatory T cell function through cooperation with NFAT. Cell. (2006) 126:375–87. doi: 10.1016/j.cell.2006.05.042 16873067

[89] Ono M Yaguchi H Ohkura N Kitabayashi I Nagamura Y Nomura T. Foxp3 controls regulatory T-cell function by interacting with AML1/Runx1. Nature. (2007) 446:685–9. doi: 10.1038/nature05673 17377532

[90] Lakshmi Narendra B Eshvendar Reddy K Shantikumar S Ramakrishna S. Immune system: a double-edged sword in cancer. Inflammation Res. (2013) 62:823–34. doi: 10.1007/s00011-013-0645-9 23868500

[91] Magg T Mannert J Ellwart JW Schmid I Albert MH. Subcellular localization of FOXP3 in human regulatory and nonregulatory T cells. Eur J Immunol. (2012) 42:1627–38. doi: 10.1002/eji.201141838 22678915

[92] Grimmig T Kim M Germer CT Gasser M Waaga-Gasser AM. The role of FOXP3 in disease progression in colorectal cancer patients. Oncoimmunology. (2013) 2:e24521. doi: 10.4161/onci.24521 23894712 PMC3716747

[93] Najafi M Farhood B Mortezaee K. Contribution of regulatory T cells to cancer: A review. J Cell Physiol. (2019) 234:7983–93. doi: 10.1002/jcp.v234.6 30317612

[94] Wang S Wu J Ren J Vlantis AC Li M-Y Liu SYW. MicroRNA-125b interacts with foxp3 to induce autophagy in thyroid cancer. Mol Ther. (2018) 26:2295–303. doi: 10.1016/j.ymthe.2018.06.015 PMC6127503 30005868

[95] Rudra D deRoos P Chaudhry A Niec RE Arvey A Samstein RM. Transcription factor Foxp3 and its protein partners form a complex regulatory network. Nat Immunol. (2012) 13:1010–9. doi: 10.1038/ni.2402 PMC3448012 22922362

[96] Hu H Djuretic I Sundrud MS Rao A. Transcriptional partners in regulatory T cells: Foxp3, Runx and NFAT. Trends Immunol. (2007) 28:329–32. doi: 10.1016/j.it.2007.06.006 17618833

[97] Wang Y Su MA Wan YY. An essential role of the transcription factor GATA-3 for the function of regulatory T cells. Immunity. (2011) 35:337–48. doi: 10.1016/j.immuni.2011.08.012 PMC3182399 21924928

[98] Konopacki C Pritykin Y Rubtsov Y Leslie CS Rudensky AY. Transcription factor Foxp1 regulates Foxp3 chromatin binding and coordinates regulatory T cell function. Nat Immunol. (2019) 20:232–42. doi: 10.1038/s41590-018-0291-z PMC7534899 30643266

[99] Jiang G Ma S Wei Y Wu Y Yu X Liu H. The prevalence and distribution of Th17 and Tc17 cells in patients with thyroid tumor. Immunol Lett. (2014) 162:68–73. doi: 10.1016/j.imlet.2014.07.005 25068436

[100] Bailey SR Nelson MH Himes RA Li Z Mehrotra S Paulos CM. Th17 cells in cancer: the ultimate identity crisis. Front Immunol. (2014) 5:276. doi: 10.3389/fimmu.2014.00276 24987392 PMC4060300

[101] Xie Y Sheng W Xiang J Ye Z Yang J. Interleukin-17F suppresses hepatocarcinoma cell growth via inhibition of tumor angiogenesis. Cancer Invest. (2010) 28:598–607. doi: 10.3109/07357900903287030 20210523

[102] Zeng R Lyu Y Zhang G Shou T Wang K Niu H. Positive effect of RORγt on the prognosis of thyroid papillary carcinoma patients combined with Hashimoto's thyroiditis. Am J Transl Res. (2018) 10:3011–24. PMC6220227 30416647

[103] Zhang X Munegowda MA Yuan J Wei Y Xiang J. Optimal TLR9 signal converts tolerogenic CD4-8- DCs into immunogenic ones capable of stimulating antitumor immunity via activating CD4+ Th1/Th17 and NK cell responses. J Leukoc Biol. (2010) 88:393–403. doi: 10.1189/jlb.0909633 20466823

[104] Azzazene D Al Thawadi H Al Farsi H Besbes S Geyl C Mirshahi S. Plasma endothelial protein C receptor influences innate immune response in ovarian cancer by decreasing the population of natural killer and TH17 helper cells. Int J Oncol. (2013) 43:1011–8. doi: 10.3892/ijo.2013.2021 PMC3829768 23877403

[105] Gogali F Paterakis G Rassidakis GZ Liakou CI Liapi C. CD3(-)CD16(-)CD56(bright) immunoregulatory NK cells are increased in the tumor microenvironment and inversely correlate with advanced stages in patients with papillary thyroid cancer. Thyroid. (2013) 23:1561–8. doi: 10.1089/thy.2012.0560 23721357

[106] Wennerberg E Pfefferle A Ekblad L Yoshimoto Y Kremer V Kaminskyy VO. Human anaplastic thyroid carcinoma cells are sensitive to NK cell-mediated lysis via ULBP2/5/6 and chemoattract NK cells. Clin Cancer Res. (2014) 20:5733–44. doi: 10.1158/1078-0432.CCR-14-0291 25212604

[107] Wang L Yi T Kortylewski M Pardoll DM Zeng D Yu H. IL-17 can promote tumor growth through an IL-6-Stat3 signaling pathway. J Exp Med. (2009) 206:1457–64. doi: 10.1084/jem.20090207 PMC2715087 19564351

[108] Guarino V Castellone MD Avilla E Melillo RM. Thyroid cancer and inflammation. Mol Cell Endocrinol. (2010) 321(1):94–102. doi: 10.1016/j.mce.2009.10.003 19835928

[109] Okayasu I. The relationship of lymphocytic thyroiditis to the development of thyroid carcinoma. Endocr Pathol. (1997) 8:225–30. doi: 10.1007/BF02738789 12114726

[110] Kebebew E Treseler PA Ituarte PH Clark OH. Coexisting chronic lymphocytic thyroiditis and papillary thyroid cancer revisited. World J Surg. (2001) 25:632–7. doi: 10.1007/s002680020165 11369991

[111] Ugolini C Basolo F Proietti A Vitti P Elisei R Miccoli P. Lymphocyte and immature dendritic cell infiltrates in differentiated, poorly differentiated, and undifferentiated thyroid carcinoma. Thyroid. (2007) 17:389–93. doi: 10.1089/thy.2006.0306 17542668

[112] Carvalho DFG Zanetti BR Miranda L Hassumi-Fukasawa MK Miranda-Camargo F Crispim JCO. High IL-17 expression is associated with an unfavorable prognosis in thyroid cancer. Oncol Lett. (2017) 13:1925–31. doi: 10.3892/ol.2017.5638 PMC5403242 28454345

[113] Jeon S-H Chae B-C Kim H-A Seo G-Y Seo D-W Chun G-T. Mechanisms underlying TGF-beta1-induced expression of VEGF and Flk-1 in mouse macrophages and their implications for angiogenesis. J Leukoc Biol. (2007) 81:557–66. doi: 10.1189/jlb.0806517 17053163

[114] Langowski JL Zhang X Wu L Mattson JD Chen T Smith K. IL-23 promotes tumour incidence and growth. Nature. (2006) 442:461–5. doi: 10.1038/nature04808 16688182

[115] Chizzolini C Chicheportiche R Alvarez M de Rham C Roux-Lombard P Ferrari-Lacraz S. Prostaglandin E2 synergistically with interleukin-23 favors human Th17 expansion. Blood. (2008) 112:3696–703. doi: 10.1182/blood-2008-05-155408 PMC2572797 18698005

[116] Yang B Kang H Fung A Zhao H Wang T Ma D. The role of interleukin 17 in tumour proliferation, angiogenesis, and metastasis. Mediators Inflamm. (2014) 2014:623759. doi: 10.1155/2014/623759 25110397 PMC4119694

[117] Mizutani K Sud S McGregor NA Martinovski G Rice BT Craig MJ. The chemokine CCL2 increases prostate tumor growth and bone metastasis through macrophage and osteoclast recruitment. Neoplasia. (2009) 11:1235–42. doi: 10.1593/neo.09988 PMC2767225 19881959

[118] Maj T Wang W Crespo J Zhang H Wang W Wei S. Oxidative stress controls regulatory T cell apoptosis and suppressor activity and PD-L1-blockade resistance in tumor. Nat Immunol. (2017) 18:1332–41. doi: 10.1038/ni.3868 PMC5770150 29083399

[119] Saito S Nakashima A Shima T Ito M. Th1/Th2/Th17 and regulatory T-cell paradigm in pregnancy. Am J Reprod Immunol. (2010) 63:601–10. doi: 10.1111/j.1600-0897.2010.00852.x 20455873

[120] Deng Y Wang Z Chang C Lu L Lau CS Lu Q. Th9 cells and IL-9 in autoimmune disorders: Pathogenesis and therapeutic potentials. Hum Immunol. (2017) 78:120–8. doi: 10.1016/j.humimm.2016.12.010 28040536

[121] Yang XO Nurieva R Martinez GJ Kang HS Chung Y Pappu BP. Molecular antagonism and plasticity of regulatory and inflammatory T cell programs. Immunity. (2008) 29:44–56. doi: 10.1016/j.immuni.2008.05.007 18585065 PMC2630532

[122] Downs-Canner S Berkey S Delgoffe GM Edwards RP Curiel T Odunsi K. Suppressive IL-17A+Foxp3+ and ex-Th17 IL-17AnegFoxp3+ Treg cells are a source of tumour-associated Treg cells. Nat Commun. (2017) 8:14649. doi: 10.1038/ncomms14649 28290453 PMC5355894

[123] Phillips JD Knab LM Blatner NR Haghi L DeCamp MM Meyerson SL. Preferential expansion of pro-inflammatory Tregs in human non-small cell lung cancer. Cancer Immunol Immunother. (2015) 64:1185–91. doi: 10.1007/s00262-015-1725-1 PMC4891203 26047578

[124] Voo KS Wang Y-H Santori FR Boggiano C Wang Y-H Arima K. Identification of IL-17-producing FOXP3+ regulatory T cells in humans. Proc Natl Acad Sci U S A. (2009) 106:4793–8. doi: 10.1073/pnas.0900408106 PMC2653560 19273860

[125] Kryczek I Wu K Zhao E Wei S Vatan L Szeliga W. IL-17+ regulatory T cells in the microenvironments of chronic inflammation and cancer. J Immunol. (2011) 186:4388–95. doi: 10.4049/jimmunol.1003251 21357259

[126] Su X Ye J Hsueh EC Zhang Y Hoft DF Peng G. Tumor microenvironments direct the recruitment and expansion of human Th17 cells. J Immunol. (2010) 184:1630–41. doi: 10.4049/jimmunol.0902813 20026736

[127] Ye J Su X Hsueh EC Zhang Y Koenig JM Hoft DF. Human tumor-infiltrating Th17 cells have the capacity to differentiate into IFN-γ+ and FOXP3+ T cells with potent suppressive function. Eur J Immunol. (2011) 41:936–51. doi: 10.1002/eji.201040682 21381020

[128] Noack M Miossec P. Th17 and regulatory T cell balance in autoimmune and inflammatory diseases. Autoimmun Rev. (2014) 13:668–77. doi: 10.1016/j.autrev.2013.12.004 24418308

[129] Fasching P Stradner M Graninger W Dejaco C Fessler J. Therapeutic potential of targeting the th17/treg axis in autoimmune disorders. Molecules. (2017) 22. doi: 10.3390/molecules22010134 PMC6155880 28098832

[130] Samson M Audia S Janikashvili N Ciudad M Trad M Fraszczak J. Brief report: inhibition of interleukin-6 function corrects Th17/Treg cell imbalance in patients with rheumatoid arthritis. Arthritis Rheumatol. (2012) 64:2499–503. doi: 10.1002/art.34477 22488116

[131] Kikuchi J Hashizume M Kaneko Y Yoshimoto K Nishina N Takeuchi T. Peripheral blood CD4(+)CD25(+)CD127(low) regulatory T cells are significantly increased by tocilizumab treatment in patients with rheumatoid arthritis: increase in regulatory T cells correlates with clinical response. Arthritis Res Ther. (2015) 17:10. doi: 10.1186/s13075-015-0526-4 25604867 PMC4332922

[132] Li S Wu Z Li L Liu X. Interleukin-6 (IL-6) receptor antagonist protects against rheumatoid arthritis. Med Sci Monit. (2016) 22:2113–8. doi: 10.12659/MSM.896355 PMC4920098 27322646

[133] Maruyama T Kono K Mizukami Y Kawaguchi Y Mimura K Watanabe M. Distribution of Th17 cells and FoxP3(+) regulatory T cells in tumor-infiltrating lymphocytes, tumor-draining lymph nodes and peripheral blood lymphocytes in patients with gastric cancer. Cancer Sci. (2010) 101:1947–54. doi: 10.1111/j.1349-7006.2010.01624.x PMC11159855 20550524

[134] Zhou J Li X Wu X Zhang T Zhu Q Wang X. Exosomes released from tumor-associated macrophages transfer miRNAs that induce a treg/th17 cell imbalance in epithelial ovarian cancer. Cancer Immunol Res. (2018) 6:1578–92. doi: 10.1158/2326-6066.CIR-17-0479 30396909

[135] Duan M-C Han W Jin P-W Wei Y-P Wei Q Zhang L-M. Disturbed th17/treg balance in patients with non-small cell lung cancer. Inflammation. (2015) 38:2156–65. doi: 10.1007/s10753-015-0198-x 26077695

[136] Li L Yang C Zhao Z Xu B Zheng M Zhang C. Skewed T-helper (Th)1/2- and Th17/T regulatory−cell balances in patients with renal cell carcinoma. Mol Med Rep. (2015) 11:947–53. doi: 10.3892/mmr.2014.2778 PMC4262517 25352158

[137] Zhang W Hou F Zhang Y Tian Y Jiao J Ma D. Changes of Th17/Tc17 and Th17/Treg cells in endometrial carcinoma. Gynecol Oncol. (2014) 132:599–605. doi: 10.1016/j.ygyno.2013.12.036 24388919

[138] Lin W Niu Z Zhang H Kong Y Wang Z Yang X. Imbalance of Th1/Th2 and Th17/Treg during the development of uterine cervical cancer. Int J Clin Exp Pathol. (2019) 12:3604–12. PMC6949808 31934210

[139] Lin W Zhang H-L Niu Z-Y Wang Z Kong Y Yang X-S. The disease stage-associated imbalance of Th1/Th2 and Th17/Treg in uterine cervical cancer patients and their recovery with the reduction of tumor burden. BMC Womens Health. (2020) 20:126. doi: 10.1186/s12905-020-00972-0 32552719 PMC7301485

[140] Chen Z Ding J Pang N Du R Meng W Zhu Y. The Th17/Treg balance and the expression of related cytokines in Uygur cervical cancer patients. Diagn Pathol. (2013) 8:61. doi: 10.1186/1746-1596-8-61 23587428 PMC3640920

[141] Wang X Wang L Mo Q Dong Y Wang G Ji A. Changes of Th17/Treg cell and related cytokines in pancreatic cancer patients. Int J Clin Exp Pathol. (2015) 8:5702–8. PMC4503155 26191284

[142] Lan Y-T Fan X-P Fan Y-C Zhao J Wang K. Change in the Treg/Th17 cell imbalance in hepatocellular carcinoma patients and its clinical value. Med (Baltimore). (2017) 96:e7704. doi: 10.1097/MD.0000000000007704 PMC5556221 28796055

[143] Liu C Tong Z Tan J Xin Z. Analysis of Treg/Th17 cells in patients with tongue squamous cell carcinoma. Exp Ther Med. (2019) 18:2187–93. doi: 10.3892/etm.2019.7814 PMC6704530 31452709

[144] Wang L Zhang Y Xie F. T-regulatory cell/T helper 17 cell imbalance functions as prognostic biomarker of oral squamous cell carcinoma - CONSORT. Med (Baltimore). (2020) 99:e23145. doi: 10.1097/MD.0000000000023145 PMC7717799 33285688

[145] Huo W Liu Y Lei Y Zhang Y Huang Y Mao Y. Imbalanced spinal infiltration of Th17/Treg cells contributes to bone cancer pain via promoting microglial activation. Brain Behav Immun. (2019) 79:139–51. doi: 10.1016/j.bbi.2019.01.024 30685532

[146] Wu H Zheng X Dong L Li C Zhang M Wang G. Pir-B inhibits the DC function and disturbs the Th17/Treg balance in lung cancer murine model. Oncotarget. (2017) 8:114710–21. doi: 10.18632/oncotarget.21763 PMC5777726 29383114

[147] Sasidharan Nair V Elkord E. Immune checkpoint inhibitors in cancer therapy: a focus on T-regulatory cells. Immunol Cell Biol. (2018) 96:21–33. doi: 10.1111/imcb.2018.96.issue-1 29359507

[148] Peggs KS Quezada SA Chambers CA Korman AJ Allison JP. Blockade of CTLA-4 on both effector and regulatory T cell compartments contributes to the antitumor activity of anti-CTLA-4 antibodies. J Exp Med. (2009) 206:1717–25. doi: 10.1084/jem.20082492 PMC2722174 19581407

[149] Okazaki T Chikuma S Iwai Y Fagarasan S Honjo T. A rheostat for immune responses: the unique properties of PD-1 and their advantages for clinical application. Nat Immunol. (2013) 14:1212–8. doi: 10.1038/ni.2762 24240160

[150] Dighe AS Richards E Old LJ Schreiber RD. Enhanced in *vivo* growth and resistance to rejection of tumor cells expressing dominant negative IFN gamma receptors. Immunity. (1994) 1:447–56. doi: 10.1016/1074-7613(94)90087-6 7895156

[151] Brahmer JR Drake CG Wollner I Powderly JD Picus J Sharfman WH. Phase I study of single-agent anti-programmed death-1 (MDX-1106) in refractory solid tumors: safety, clinical activity, pharmacodynamics, and immunologic correlates. J Clin Oncol. (2023) 41:715–23. doi: 10.1200/JCO.22.02270 36706735

[152] Fallahi P Ferrari SM Galdiero MR Varricchi G Elia G Ragusa F. Molecular targets of tyrosine kinase inhibitors in thyroid cancer. Semin Cancer Biol. (2022) 79:180–96. doi: 10.1016/j.semcancer.2020.11.013 33249201

[153] Fallahi P Mazzi V Vita R Ferrari SM Materazzi G Galleri D. New therapies for dedifferentiated papillary thyroid cancer. Int J Mol Sci. (2015) 16:6153–82. doi: 10.3390/ijms16036153 PMC4394525 25789503

